# Ipsilateral Double Parathyroid Adenomas Causing Primary Hyperparathyroidism: A Rare Case

**DOI:** 10.7759/cureus.114051

**Published:** 2026-08-06

**Authors:** Nathaniel G Blanchard, Marine Bolliet, Ramachandra Kolachalam

**Affiliations:** 1 Department of Surgery, Henry Ford Providence Southfield Hospital - Michigan State University College of Osteopathic Medicine, Southfield, USA

**Keywords:** double parathyroid adenoma, head-neck surgery, intraoperative pth monitoring, parathyroidectomy, preoperative localization, primary hyperparathyroidism

## Abstract

Double parathyroid adenomas represent an uncommon but well-recognized subset of primary hyperparathyroidism. Awareness of this possibility is essential, as failure to identify multigland involvement preoperatively may result in intraoperative uncertainty, incomplete resection, and persistent disease. Despite advances in localization techniques, the sensitivity of commonly used imaging modalities - including ultrasonography, technetium-99m sestamibi scintigraphy, and four-dimensional computed tomography - remains limited in detecting double adenomas. We present the case of a patient with primary hyperparathyroidism, who had two left-sided parathyroid adenomas identified on preoperative imaging and confirmed intraoperatively. This report aims to highlight ipsilateral double adenoma disease as a rare but clinically significant entity. In addition, we review adjunctive preoperative risk stratification models and intraoperative biochemical monitoring strategies that may improve recognition of multigland disease and reduce the need for extensive bilateral neck exploration.

## Introduction

Primary hyperparathyroidism (PHPT) is an endocrine disorder characterized by autonomous overproduction of parathyroid hormone (PTH) from one or more parathyroid glands. Excess PTH results in hypercalcemia and its well-recognized clinical manifestations, including nephrolithiasis, skeletal demineralization and bone pain, gastrointestinal disturbances, and neuropsychiatric symptoms [1]. In the United States, the annual incidence of PHPT is estimated at approximately one per 100,000 individuals, with women three to four times more likely to develop PHPT compared to men [1].

The etiologies of PHPT are heterogeneous; however, most cases fall into several principal categories: single parathyroid adenoma, double adenomas, multigland hyperplasia, parathyroid carcinoma, and hereditary or syndromic causes [1]. Single adenomas account for approximately 85-90% of cases, whereas double adenomas comprise a substantially smaller proportion, estimated at 1.7-9% [2,3]. Ipsilateral involvement in double adenomas constitutes a further level of rarity, reported in approximately 29.6%-34% of cases compared with contralateral involvement [4,5].

We present the case of a 59-year-old woman with a history of hypercalcemia and elevated PTH levels who underwent parathyroidectomy for primary hyperparathyroidism. Preoperative localization studies suggested two distinct ipsilateral parathyroid adenomas. This report describes her clinical course, operative and postoperative management, and discusses the diagnostic and surgical considerations associated with this uncommon presentation.

## Case presentation

A 59-year-old woman presented with an eight-month history of progressive fatigue and lethargy. She also reported neurocognitive symptoms, including mental fogginess, intermittent confusion, and impaired concentration. Routine laboratory evaluation demonstrated hypercalcemia and elevated PTH level, which are findings consistent with primary hyperparathyroidism.

Diagnostic assessment

During the diagnostic evaluation, laboratory studies confirmed hypercalcemia, with an ionized calcium level of 1.54 mmol/L, and a markedly elevated PTH level of 264 pg/mL (Table 1).

**Table 1:** Patient laboratory studies

Parameter	Patient Value	Reference Range
Ionized calcium level	1.54 mmol/L	1.13-1.32 mmol/L
Parathyroid hormone (PTH)	264 pg/mL	15-65 pg/mL

**Figure 1:**
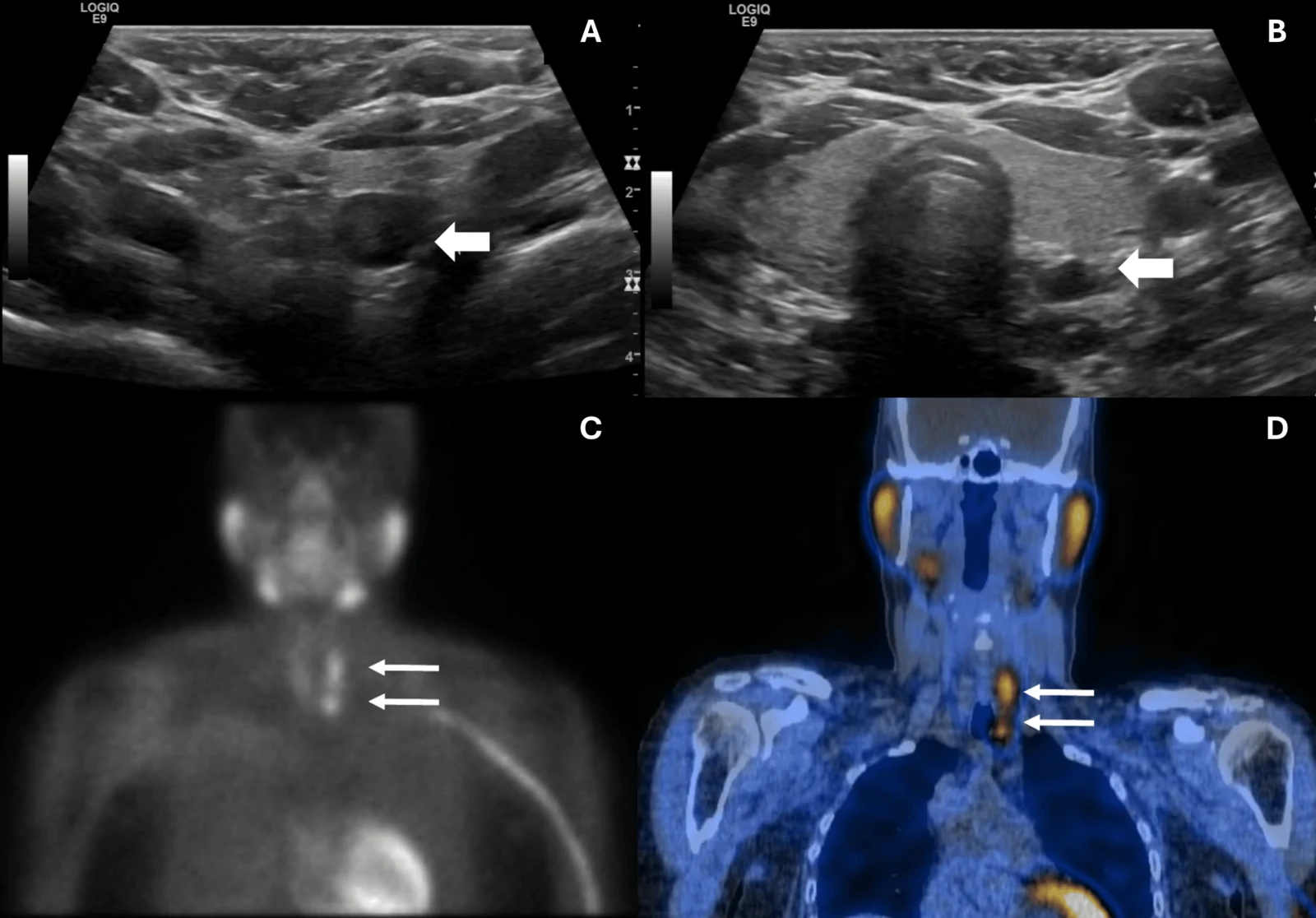
Preoperative imaging demonstrating left-sided double parathyroid adenomas (A) Transverse neck ultrasound demonstrating a left inferior parathyroid adenoma (arrow). (B) Transverse neck ultrasound demonstrating a left superior parathyroid adenoma (arrow). (C) Technetium-99m sestamibi (Tc-99m MIBI) scintigraphy, grayscale image, demonstrating radiotracer uptake corresponding to the left-sided double parathyroid adenomas (arrow). (D) Color-enhanced Tc-99m MIBI scintigraphy demonstrating radiotracer uptake corresponding to the left-sided double parathyroid adenomas (arrow).

Cervical ultrasonography identified a 1.8 cm extrathyroidal nodule located medial to the inferior pole of the left thyroid lobe, highly suspicious for a parathyroid adenoma (Figure 1A). A second extrathyroidal lesion was also visualized on the left side, situated posterior to the mid-portion of the thyroid gland and slightly more lateral in position 9 (Figure 1B). This lesion was initially interpreted as a possible lymph node.

Subsequent nuclear medicine imaging with a technetium-99m sestamibi scan demonstrated two discrete foci of radiotracer uptake at the levels of the mid and inferior portions of the left thyroid lobe, respectively (Figures 1C-1D). Both areas of increased uptake were interpreted as consistent with parathyroid adenomas, supporting the diagnosis of ipsilateral double adenomas.

Treatment

The patient was scheduled for a left-sided parathyroidectomy with intraoperative recurrent laryngeal nerve monitoring. A baseline intraoperative PTH level, obtained prior to gland manipulation, was 264 pg/mL.

The left superior parathyroid gland was identified and excised first. The specimen weighed 1.20 g. Ten minutes following excision, the intraoperative PTH level decreased to 153 pg/mL, but was not sufficient for a 50% reduction from the preoperative value. Attention was then turned to the previously seen left inferior parathyroid on imaging, which was subsequently identified and removed. This specimen weighed 0.93 g. Five minutes after excision of the inferior gland - and 15 minutes after removal of the superior gland - the PTH level declined to 50 pg/mL, now representing a greater than 50% reduction from baseline and meeting criteria for adequate biochemical response.

Both specimens were submitted for histopathologic evaluation, which confirmed the diagnosis of two parathyroid adenomas. The patient's postoperative course was uncomplicated. She was discharged home on postoperative day one.

## Discussion

The clinical importance of accurately identifying double parathyroid adenomas cannot be overstated. Failure to recognize and excise all hyperfunctioning parathyroid tissue may result in persistent or recurrent primary hyperparathyroidism. Moreover, accurate preoperative localization of multiple adenomas has direct implications for operative strategy, influencing the decision between a focused minimally invasive parathyroidectomy and a more comprehensive bilateral neck exploration.

Ipsilateral double adenomas represent an uncommon subset of primary hyperparathyroidism. Although double adenomas account for approximately 1-9% of all cases of primary hyperparathyroidism, their anatomical distribution varies considerably [2]. In a study of 27 patients with double adenomas, six distinct configurations were identified; 70.4% were contralateral, and 29.6% were ipsilateral [5]. Notably, only 11.1% of cases involved two left-sided adenomas [5]. Similar findings were reported by Kandil et al., who observed contralateral involvement in 66% and ipsilateral involvement in 34% of patients with double adenomas [4]. Collectively, these data underscore both the heterogeneity of double adenoma presentations and the relative rarity of ipsilateral, particularly left-sided, disease.

In the present case, preoperative workup supported the diagnosis of true double adenoma disease. During the diagnostic evaluation for primary hyperparathyroidism, neck ultrasonography identified a suspicious parathyroid lesion. Subsequent technetium-99m sestamibi scintigraphy demonstrated two distinct foci consistent with adenomas. Normal parathyroid glands typically weigh between 0.02 g and 0.04 g, whereas adenomatous glands average approximately 1 g, with rare cases exceeding 3.5 g [6]. The excised left superior and inferior glands in this patient weighed 1.20 g and 0.93 g, respectively, both well within the expected range for adenomas. The relatively small difference in weight between the two glands (0.27 g) argues against the presence of a single dominant adenoma with a second, incidentally enlarged but nonfunctioning gland. Histopathologic examination of both specimens confirmed parathyroid tissue consistent with adenomas, further substantiating the diagnosis.

Although both adenomas were successfully identified preoperatively on sestamibi imaging in this case, this level of concordance is not consistently achieved. For example, Tzikos et al. described a patient who underwent focused parathyroidectomy after preoperative imaging suggested single-gland disease, only to have a second adenoma identified intraoperatively [3]. In a study by de Gregorio et al., the reported sensitivities for detecting double adenoma disease were 41.8% for ultrasonography, 34.5% for technetium-99m sestamibi scanning, and 64.3% for four-dimensional computed tomography [7]. These limitations in preoperative imaging highlight the challenges inherent in identifying multigland disease and reinforce the importance of adjunctive risk stratification tools and intraoperative biochemical assessment.

Two commonly cited preoperative predictive models are the Wisconsin Index (WIN) and the Calcium Parathyroid Hormone and Ultrasound Score (CaPTHUS) score [8,9]. The WIN is calculated as the product of preoperative serum calcium and PTH levels (WIN = Ca²⁺ × PTH) and stratifies patients into low (<800), medium (801-1,600), and high (>1,600) risk categories for multigland disease [8]. Higher scores are associated with an increased likelihood of multigland involvement. In contrast, the CaPTHUS score estimates the probability of single-gland disease using five criteria: serum calcium >12 mg/dL, PTH greater than twice the upper limit of normal, positive sestamibi scan for a single gland, positive ultrasound demonstrating a single enlarged gland, and concordance between imaging modalities [9]. One point is assigned for each criterion met. In a retrospective study of 1,421 patients, Elfenbein et al. demonstrated that a CaPTHUS score ≥3 had a 91% positive predictive value for single-gland disease [9]. This implies that nearly 9% of patients with this score may harbor multigland pathology, underscoring the need for continued intraoperative vigilance.

Intraoperatively, the "Miami criterion" remains a widely accepted standard for confirming adequate resection. This criterion requires a greater than 50% reduction in PTH from the highest pre-excision level following gland removal [10]. Failure to achieve this threshold should prompt further exploration for additional hyperfunctioning tissue. In the present case, sequential excision of both glands, combined with intraoperative PTH monitoring, demonstrated an appropriate biochemical response only after removal of both lesions, further confirming the presence of dual adenomas.

## Conclusions

This case highlights ipsilateral double parathyroid adenomas as a rare but clinically significant cause of primary hyperparathyroidism. Awareness of this entity - along with careful preoperative imaging and risk stratification and rigorous intraoperative biochemical monitoring - is essential to guide operative management, minimize the risk of persistent disease, and optimize patient outcomes.
